# Parenting and psychosis: An experience sampling methodology study investigating the inter‐relationship between stress from parenting and positive psychotic symptoms

**DOI:** 10.1111/bjc.12389

**Published:** 2022-08-08

**Authors:** Jessica Radley, Jane Barlow, Louise C. Johns

**Affiliations:** ^1^ Department of Psychiatry University of Oxford Warneford Hospital Oxford UK; ^2^ Department of Social Policy and Intervention University of Oxford Oxford UK

**Keywords:** ESM, experience sampling, longitudinal, parenting, psychosis, stress

## Abstract

**Objectives:**

There is a strong association between stress and psychotic symptoms, and this study examined the bidirectional nature of this relationship in parents with psychosis, with negative affect as a mediator and a range of other psychosocial factors included as covariates. It also examined whether stress from parenting had a larger impact on psychosis than non‐parenting stress.

**Design:**

The study used a within‐participants repeated measures design, using experience sampling methodology (ESM). ESM is a self‐report surveying technique completed over an intensive longitudinal period. Participants completed six surveys a day, for 10 days.

**Methods:**

Thirty‐five participants with psychosis who were a parent to a child between the ages of 2 and 16 took part. Study phones alerted participants to complete surveys by beeping at semi‐random intervals over 10 days. Multi‐level modelling was used with surveys at Level‐1 and participants at Level‐2. Predictor variables were time‐lagged in order to infer directionality.

**Results:**

Parenting stress was found to predict psychotic symptoms, and this relationship was mediated by negative affect. The reverse direction was also confirmed. Few of the additional psychosocial factors were found to have a significant impact on the models' estimations. Parenting stress was not found to have a larger impact on psychosis than other sources of stress.

**Conclusions:**

This study provides further evidence of the bidirectional relationship between stress and psychosis in the context of parenting. Further research should explore if parenting stress plays a unique role in predicting psychotic symptoms by comparing parents and non‐parents with psychosis.

**Practitioner points**

- Parenting is stressful. Practitioners working with parents with psychosis should be aware of the role of stress in exacerbating psychotic symptoms.
- Practitioners should work with parents with psychosis to identify their daily stressors and the significance they attribute to them.
- Low mood is an important mediator of the relationship between stress and psychosis, and is therefore another essential factor to address when working with parents with psychosis.

## INTRODUCTION

Between 36% and 44% of people diagnosed with a psychotic disorder are parents (Campbell et al., 2012; Radley et al., 2022). Parenting is a valued role for people with psychosis (Chernomas et al., 2000), but research suggests that the symptoms associated with psychosis may make it more difficult for parents to cope with the daily demands of having children. For example, delusions and hallucinations can preoccupy a parent, leaving them unable to focus on their child's needs, while the negative symptoms of psychosis and sedation from antipsychotic medication can mean the parent withdraws emotionally from their children (Seeman, 2015; Snellen et al., 1999; Somers, 2007). These difficulties can lead to an overly permissive or disciplinary parenting style (Johnson et al., 2018; Oyserman et al., 2005). In turn, parents with severe mental illness, such as psychosis, are more likely to have children with greater behavioural and psychological needs than is typical in a general population (Campbell et al., 2012). They are also more likely than parents without severe mental illness to be socially disadvantaged, unemployed, single and have less social support (Benjet et al., 2003; Fusar‐Poli et al., 2015; Killaspy et al., 2014; Owen et al., 2016), all of which act as risk factors for poor parenting, and poor mental health prognosis for both the parents and the children in these families (Abel et al., 2005; Campbell et al., 2018; Mowbray et al., 2005; Riches et al., 2019).

The stress‐vulnerability hypothesis suggests that in individuals with a pre‐existing vulnerability, psychosis can occur as a result of stressful life events (Myin‐Germeys & van Os, 2007; Zubin & Spring, 1977). Many studies have demonstrated a relationship between stress and increases in psychotic symptoms (Klippel et al., 2017; Palmier‐Claus et al., 2012; Reininghaus et al., 2016), with more recent research showing that daily stress can precede daily psychotic symptoms (Klippel et al., 2018; So et al., 2018; Vaessen et al., 2019) across the continuum of psychosis (at risk, early and chronic). The majority of parents with psychosis have had their child before their first psychotic episode (Caton et al., 1999; Mowbray et al., 2005), and parenting‐related stress could be both the cause of a first psychotic episode, and contribute to the repeat onset of psychotic symptoms.

This study aimed to investigate the role of stress from parenting in the exacerbation of psychotic symptoms by using experience sampling methodology (ESM) to measure daily fluctuations in both. ESM is a self‐report diary technique that allows researchers to measure momentary experiences in a real‐life setting. Participants are typically given a device to keep with them during their daily lives, which alerts them to complete surveys on their current experiences. This method is better than typical laboratory‐based studies for sampling mood, symptoms and contextual appraisals since it does not rely on recall and has high ecological validity (Myin‐Germeys et al., 2018; Palmier‐Claus et al., 2019). It has also been shown to be an acceptable and feasible means of measuring the daily lives of people with psychosis (Oorschot et al., 2009; Vachon et al., 2019).

This study built upon the large body of ESM research looking at the relationship between stress and psychotic symptoms in people with psychosis on a moment‐to‐moment basis (e.g. Collip et al., 2011; Cristóbal‐Narváez et al., 2016; Klippel et al., 2017; Lataster et al., 2013; Myin‐Germeys et al., 2001; Palmier‐Claus et al., 2012; Reininghaus et al., 2016; So et al., 2018; Vaessen et al., 2019), but maintained a specific focus on stress due to parenting. We hypothesized that parenting would have more of an impact on psychotic symptoms than other sources of stress in participants' lives, which has also been hypothesized in research looking at the impact of stress on substance use in parents (Rutherford & Mayes, 2019).

Individuals who experience psychosis have higher stress sensitivity, that is have a stronger negative emotional response to stressful life events when compared to a group without psychosis (DeVylder et al., 2013; Reininghaus et al., 2016), and even when compared to people with affective diagnoses (Myin‐Germeys et al., 2003). This has led to negative affect consistently being included in models as an important mediator of the relationship between stress and psychotic symptoms (Klippel et al., 2017; Lataster et al., 2013; van der Steen et al., 2017). This study therefore included negative affect as a mediator when modelling the effects of stress from parenting on psychotic symptoms.

**FIGURE 1:**
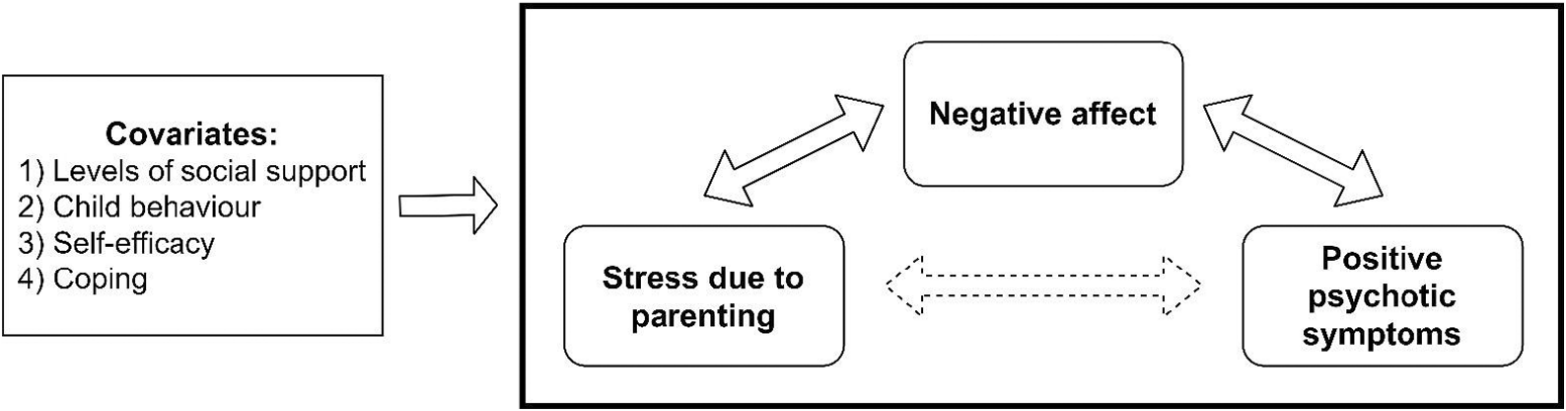
Model diagram

In addition, a range of factors have been identified as being important in moderating outcomes for parents with mental illness, including child behaviour (Campbell et al., 2012), coping strategies (Ered et al., 2017), parent gender (Ranning et al., 2016; Reedtz et al., 2019), parenting self‐efficacy (Oyserman et al., 2004) and social support (Kahng et al., 2008). This study therefore also examined the influence of these factors on the relationship between psychosis and stress due to parenting. Finally, there is still debate regarding the direction of causality between stress and psychosis (e.g. van der Steen et al., 2017), and therefore time‐lagged variables were used to investigate directionality. These aims are detailed in the model shown in Figure 1.

Hypothesis 1 Stress due to parenting will increase positive psychotic symptoms. This effect will be mediated by negative affect.

Hypotheses 2 The following items will improve the model's estimation when included as covariates: (1) levels of social support, (2) child behaviour, (3) parenting self‐efficacy, (4) coping strategies.

Hypothesis 3 Stress due to parenting will have a larger impact on positive psychotic symptoms than general stress.

Hypothesis 4 Positive psychotic symptoms will increase stress due to parenting. This effect will be mediated by negative affect.

## METHODOLOGY

### Design

This study used experience sampling methodology (ESM) in which participants completed multiple surveys over the study period. ESM is a self‐report surveying technique where multiple reports are completed during the course of a day over a pre‐determined time period (Myin‐Germeys et al., 2018; Palmier‐Claus et al., 2019). In this study, participants were surveyed six times a day over a period of 10 days, for a total of sixty times. Every participant was asked to complete the same 20–21 questions at every survey. The ESM surveys were designed using mobileQ software, and mobileQ software had been downloaded onto Nokia 2.1 phones, which were given to participants (Meers et al., 2020).

### Participants

Participants were recruited from Early Intervention in Psychosis services and Adult Mental Health Teams in Oxford Health Foundation Trust. Participants were eligible if their children were between 2 and 16 years of age, representing parents whose children were still in need of daily care from them, while avoiding specific stresses that accompany the perinatal period. Participants were also eligible if they had a case note primary diagnosis of any psychotic disorder and lived full‐time with their child. Individuals were excluded if their psychosis had been classified as follows: postpartum psychosis, drug‐induced psychosis, organic psychosis or if they did not speak English.

Participants were recruited either through contact with their care coordinator at the Trust or through a database of those who have previously given consent to be contacted for research. At the first contact, the researcher explained the study and checked whether the parent was eligible to take part. Participants were given at least 24 hours to consider whether they wanted to take part or not. Before giving written informed consent to take part, participants were given the opportunity to ask any questions they had about the project.

### Sample size

The sample size was calculated in R using the simr package at 80% power (Green & Macleod, 2016). A multi‐level model with psychosis as the outcome, and stress and negative affect as independent variables, was simulated using data from van der Steen et al. (2017). This was repeated for the three types of stress (activity, event and social), and the largest sample size returned was thirty‐two.

### Procedure

Due to the covid‐19 restrictions that were in place while planning this study, the study procedure was designed to minimize the interaction between the participant and the researcher. Using Qualtrics, the participants provided informed consent, demographic details and completed questionnaires on social support, their child's behaviour, parenting self‐efficacy and coping. They then watched a video demonstration of the ESM procedure. A researcher was on the phone with the participant while they watched the video so they could immediately ask any questions. The researcher then endeavoured to deliver the study phone to the participant's home as soon as possible after this.

The study phones were in ‘kiosk‐mode’, meaning the participant was only able to interact with the mobileQ interface (Meers et al., 2020). The ESM surveys were designed on the web‐based platform and linked to the phones via a QR code. The study phone alerted participants (beeped) six times over ten consecutive days between the hours of 7:30 am and 9:30 pm on weekdays and 9 am and 11 pm on weekends. Once the phone had beeped, the participant had 5 minutes to respond to the survey before it disappeared. Each survey included 20 or 21 questions, piloted to take 2 minutes to complete. The surveys occurred at random intervals during the day and the minimum amount of time between surveys was 1 hour. The researcher called each participant within 3 days of the start of the study in order to address any issues that had arisen. The participants were also provided with a troubleshooting document alongside the phones and contact details of a researcher, which they could use to ask any questions that arose during the study period. After the 10 days of participation, the researcher arranged a time to meet with the participant, where the participant returned the study phone and was paid £25 for participating.

The design and procedure of this research project was discussed during a Public and Patient Involvement (PPI) day with a group of parents with psychosis. Following this, changes were made to the participation fee and the hours of the day during which participants would be surveyed. The troubleshooting document was also introduced following recommendations from the PPI members at this meeting.

### Measures

#### Social support

The Medical Outcomes Study – Social Support Survey (19 items) was used to assess social support. It has four subscales: emotional/informational, tangible, affectionate and positive social interactions. It has high discriminant and concurrent validity and reliability for all of its subscales (Cronbach's alpha >.91) (Sherbourne & Stewart, 1991).

#### Child behaviour

Child behaviour was measured using the Strength and Difficulties Questionnaire (Goodman, 1997) (25 items). A total difficulties score for each participant was produced by summing four subscales: emotional problems, conduct problems, hyperactivity and peer problems. It has high concurrent validity when compared to other measures of child behaviour (Goodman, 1997) and has been shown to have good test–retest reliability (Yao et al., 2009).

If the parent had more than one child, they were asked to answer the SDQ in reference to their child whose behaviour they found the most challenging. Parents with children aged between 2 and 3 years 11 months completed the 2–4 year old version of the SDQ and parents with children aged 4 years and older, completed the 4–17 year old version.

#### Parenting self‐efficacy

The ‘Me as a Parent’ Questionnaire (Hamilton et al., 2015) (16 items) was used to measure parenting self‐efficacy. It comprises four subscales: self‐efficacy, personal agency, self‐sufficiency and self‐management. The scale has been shown to have high content validity and internal consistency (Wittkowski et al., 2017). Cronbach's alpha for the subscales ranged from .63 to .75 (Hamilton et al., 2015).

#### Coping

The Coping Self‐Efficacy Scale (26 items) was used to measure participant's self‐rated ability in using coping behaviours during stressful events. It comprises three sub‐scales: using problem‐focused coping, stopping unpleasant emotions and thoughts, and getting support from friends and family. Construct validity was confirmed through factor analyses, and the scale was shown to have high test–retest reliability and Cronbach's alpha for the subscales ranged from .80 to .91 (Chesney et al., 2006).

#### ESM items

The ESM items for negative affect and stress were taken from previous ESM studies measuring similar concepts in similar populations (Klippel et al., 2017; Myin‐Germeys et al., 2001; Palmier‐Claus et al., 2012; Reininghaus et al., 2016; Vaessen et al., 2019; van der Steen et al., 2017). Thirteen ESM items for psychosis taken from other ESM studies (Klippel et al., 2017; Lardinois et al., 2011; Myin‐Germeys et al., 2001) were presented to the PPI group, where they were refined down to eight items after members assessed the comprehensibility of each question and whether it accurately described their experience. Feedback from the PPI group also highlighted the importance of framing some of the psychosis questions positively in order to alleviate distress where possible. ESM items were either presented to participants on a sliding scale from 0 to 100 or as a multiple tick‐box question, depending on the type of question (see Table 1).

**TABLE 1:** ESM measures

Measure	ESM items
Negative affect	I feel down I feel anxious I feel insecure I feel lonely I feel guilty
Positive psychotic symptoms	I feel like I can trust people^a^ I feel safe^a^ I feel like I'm detached from reality I'm preoccupied by my thoughts I'm having difficulty expressing my thoughts I'm more sensitive than usual to the world around me I'm finding it easy to concentrate^a^ I'm hearing or seeing things that other people cannot
Activity stress	What are you doing? Tick all that are applicable [multiple‐choice question] *Parenting activity, b. Work, c. Household chores, d. Travelling, e. Leisure activity, f. Sport, g. Social contact, h. Eating/drinking, i. Something else* I would rather do something else This is difficult for me I can do this well^a^
Event stress	What was the most important event since the last beep? Tick all that are applicable [multiple‐choice question] *Parenting activity, b. Work, c. Household chores, d. Travelling, e. Leisure activity, f. Sport, g. Social contact, h. Eating/drinking, i. Something else* How pleasant was that event?
Social stress	Who are you with right now? Tick all that are applicable [multiple‐choice question] *My child/children, b. Other people I know, c. Other people I do not know, d. I'm alone* *If answer is d, no more questions. If answer is any of a‐c*: I would prefer to be alone

*Note*: All questions, except for multiple choice questions, were presented on a sliding scale from 0–100.

^a^ Items that were reverse‐coded.

Negative affect and positive psychotic symptoms were taken as the mean of the corresponding items in Table 1. Stress was measured in three ways: activity stress, event stress and social stress. Activity stress was taken as the mean of three items, and event and social stress only had one item each (see Table 1). If a participant had answered ‘I'm alone’ to the social stress multiple‐choice question, then they would not receive a score for that survey. Stress due to parenting was taken as any instances where ‘parenting activity’ or ‘my child/children’ was selected in the multiple‐choice questions (see Table 1).

### Data analysis

Any participant who completed fewer than 20 out of the 60 total surveys was excluded from the analysis, as is common practice in ESM studies (Lataster et al., 2013; Myin‐Germeys et al., 2001; van der Steen et al., 2017). Summary statistics were computed for all variables. Compliance rates were computed and any associations between missingness and variables or time‐of‐the‐day were investigated; we assumed that no associations would be found and we could therefore assume the data were ‘Missing at Random’. However, if this was violated, whereby data were found to be ‘Missing Not at Random’, data analysis still proceeded as normal and the relationship of the missingness with other variables was reported (Rubin, 1976). Due to the design of the study, missing data were present for an entire timepoint entry. If the participant was considered to have dropped‐out, whereby the majority of the missingness occurred towards the end of their participation, imputation was not conducted for this period and instead this part of their data were not used. Otherwise, an appropriate imputation method was used, for example multiple imputation for multilevel models (Grund et al., 2018).

A time‐lagged version of each ESM variable was created whereby each variable was moved one datapoint earlier in the day so that it could be compared to later surveys. This was done for all surveys except for when the timepoint was the first survey of the day. Where the timepoint is the first survey of the day, this value was made missing for the time‐lagged variable. Time‐lagged variables were referred to as *t*_−1_ and non‐time‐lagged variables were referred to as *t*_0_.

All analyses used multi‐level modelling, with Level‐1 as ‘survey/beep’ and Level‐2 as ‘participant’, and maximum likelihood estimation was employed. All models included parent age, parent gender and time‐of‐the‐day as covariates. The effect of parenting stress at *t*_−1_ on psychosis at *t*_0_ was observed. This was done separately with all three types of parenting stress – activity, event and social. Negative affect was then introduced to the model as a mediator, and confidence intervals were estimated using the Quasi‐Bayesian method. If the ACME (average causal mediation effect) was found to be statistically significant (which was defined as *p* < .02 to be certain of any effect found), then the model with negative affect was used for further analyses. If there was no effect, negative affect was not included in the model.

Then, the Level‐2 variables of social support, child behaviour, parenting self‐efficacy and coping were introduced as covariates. These Level‐2 covariates as well as gender were also investigated to see whether they moderated the effect of parenting stress on psychosis. Rather than looking at the *p*‐values of individual covariates, multiple iterations of models were compared by using a likelihood ratio test to determine which model had the best fit.

Subsequently, the opposite direction, the effect of psychosis at *t*_−1_ on parenting stress at *t*_0_, was investigated in the same way, where the three types of parenting stress were modelled separately, negative affect was investigated as a potential mediator and finally Level‐2 covariates were included in the models. All statistical analyses were conducted using R (v4.1.3).

## RESULTS

### Demographics and measures

Thirty‐five participants in total took part in this study. More participants were recruited than was deemed necessary from the sample size calculation because it was not possible to determine whether participants had completed a sufficient number of surveys to be included in the analysis until the study phones had been returned. Demographic details are presented in Table 2. The sample comprised 28 women and 7 men, and the mean age was 41 years (s.d. 6.49). Participants had between 1 and 6 children, with the average being 2. The largest ethnicity group was White British followed by Asian/Asian British. Most participants were married or had previously been married. The average age of onset of psychosis was 36.66 (s.d. 8.09) and the mean number of hospitalizations was .94 (s.d. 1.49).

**TABLE 2:** Demographics of participants

Characteristic	Participants (*n* = 35)
Age, mean (SD)	41 (6.49)
Gender, *n* (%)	
Female	28 (80%)
Male	7 (20%)
Ethnicity, *n* (%)	
White British	21 (60%)
Asian/Asian British	7 (20%)
Black/Black British	4 (11.4%)
White other	2 (5.7%)
Mixed ethnicity	1 (2.9%)
Marital status, *n* (%)	
Married	20 (57.1%)
Separated/divorced/widowed	8 (22.9%)
Single	7 (20%)
Highest school qualification, *n* (%)	
None	3 (8.6%)
Secondary school	15 (42.9%)
Undergraduate qualification	9 (25.7%)
Masters or higher	8 (22.9%)
Employment, *n* (%)	
Full time	10 (28.6%)
Part time	8 (22.9%)
Homemaker	8 (22.9%)
Self‐employed	1 (2.9%)
Unemployed	5 (14.3%)
Unable to work	3 (8.6%)
Accommodation, *n* (%)	
Renting	16 (45.8%)
Owning	14 (40%)
Other	5 (14.3%)
Age of first psychotic episode, mean (SD)	36.66 (8.09)
Number of hospitalisations, mean (SD)	.94 (1.49)
Number of children, mean (SD)	2.37 (1.46)

**FIGURE 2:**
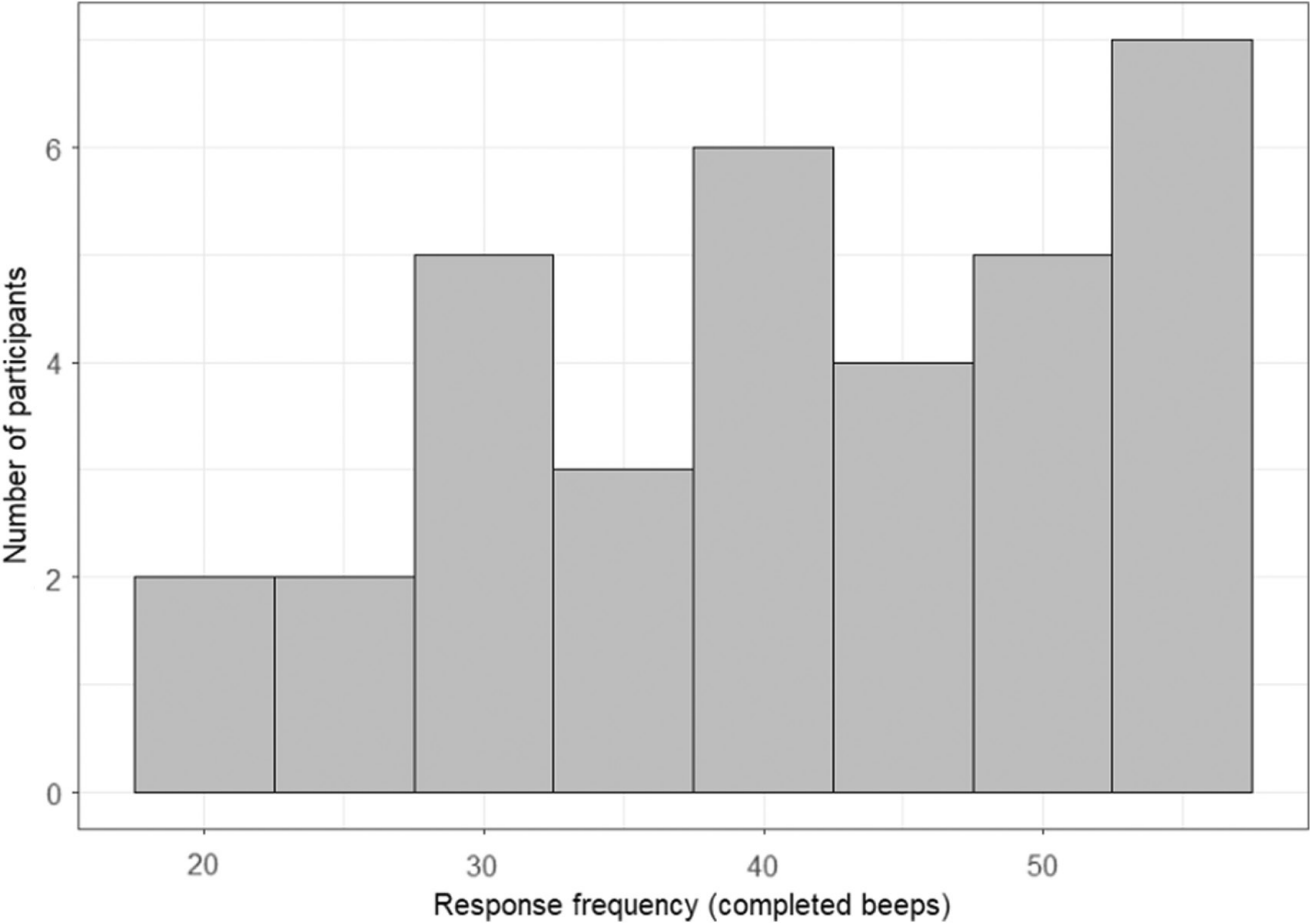
Histograms of responses to ESM surveys

Only one participant completed fewer than 20 out of the 60 total ESM surveys and they were removed before analysis began, resulting in a total of 34 participants being included in the analysis. The mean number of surveys completed was 41.2 (s.d. 11.3). Figure 2 displays the frequency of completed surveys. The means of the Level‐2 time‐invariant measures and the ESM surveys are presented in Table 3.

**TABLE 3:** Questionnaire means and standard deviations

Measure		Mean	SD	Range of measure
Time‐invariant (Level‐2) measures				
MOS – social support total score		3.52	.91	1–5
Coping self‐efficacy scale total score		5.34	1.80	0–10
Strengths and difficulties questionnaire total difficulties score		10.79	6.88	0–40
Me as a parent total score		3.71	.44	1–5
Time‐varying (Level‐1) measures				
Psychosis		26.87	22.21	0–100
Negative affect		23.21	25.74	0–100
Activity stress	All activities	26.28	21.90	0–100
	Parenting activity only	22.49	18.62	0–100
Event stress	All events	28.90	25.57	0–100
	Parenting events only	23.49	21.28	0–100
Social stress	All social situations	18.70	24.97	0–100
	Situations with children only	19.23	24.79	0–100

*Note*: *n* = 34 for all variables, except Me as a Parent total score which was completed by 31 participants.

A *t*‐test revealed that time‐of‐the‐day did not predict missingness in the psychosis variable (*t*[1239.1] = .295, *p* = .768); however, observation number (*t*[1255.3] = 4.905, *p* < .001) did, in that participants were more likely to have missed surveys towards the end of the study. Therefore, observation number was included in all models during the analysis.

Listwise deletion of missing data was used during analysis, and subsequently sensitivity analyses were performed using multiple imputation.

### Modelling

**FIGURE 3:**
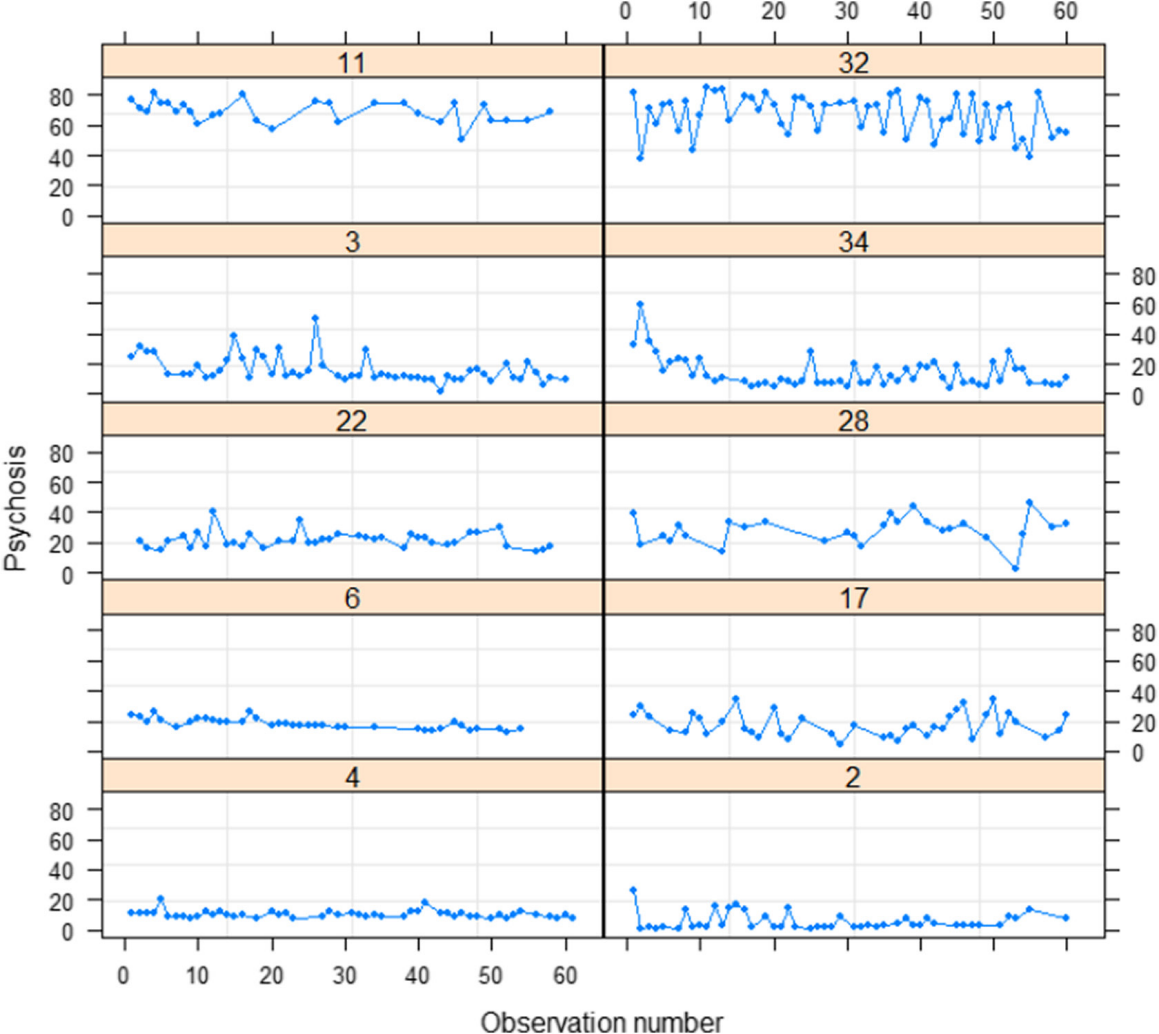
Psychosis scores for 10 participants in the sample

A multi‐level model with psychosis as the response variable and observation number as the explanatory variable was compared to an ordinary least squares model (a linear model with only one level), and a likelihood ratio test showed that the multi‐level model was superior (*X* ^*2*^[1] = 2955, *p* < .001), justifying its use. The intraclass correlation of the empty multi‐level model indicated that 90.2% of the variation in the sample is due to between‐person differences. Figure 3 presents a plot of the psychosis scores for a subset of individuals.

#### Hypothesis 1: The impact of parenting stress on positive psychotic symptoms

All three models with the three types of parenting stress (activity, event and social) at *t*_−1_ significantly predicted psychosis scores at *t*_0_:

- Parenting event stress: β(S.E.) = .09 (.03), 95% CI [.03–.15], *p* = .005
- Parenting activity stress: β(S.E.) = .16 (.04), 95% CI [.07–.24], *p* < .001
- Parenting social stress: β(S.E.) = .04 (.02), 95% CI [.01–.08], *p* = .016.

Negative affect significantly mediated the relationship between all three types of parenting stress *t*_−1_ and psychosis *t*_0_, and for event stress and social stress, the direct effect of parenting stress *t*‐_1_ on psychosis *t*_0_ became non‐significant, indicating that negative affect completely mediated this relationship (see Table 4). Therefore, negative affect was included in the modelling of psychosis with all three types of parenting stress.

**TABLE 4:** Mediation effects of negative affect *t*_−1_ on the relationship between parenting stress *t*_−1_ and psychosis *t*_0_

	Parenting event stress		Parenting activity stress		Parenting social stress	
	Estimate (95% CI)	*p*‐value	Estimate (95% CI)	*p*‐value	Estimate (95% CI)	*p*‐value
Average causal mediation effect	.03 (.01–.06)	<.001*	.05 (.02–.08)	<.001*	.02 (.01–.03)	<.001*
Average direct effect	.06 (.00–.12)	.066	.12 (.04–.20)	.014*	.03 (−.01–.06)	.154
Total effect	.09 (.03–.15)	.002*	.17 (.08–.25)	<.001*	.05 (.01–.08)	.018*
Proportion mediated	.36 (.15–1.06)	.002*	.29 (.11–.65)	<.001*	.41 (.15–1.74)	.018*

*Significance at .02 level.

Participants' mean levels of negative affect had a significant effect in all models, in that participants with higher average levels of negative affect reported higher levels of psychosis when other variables were held equal. Participants' mean levels of stress had a significant effect in two of the models. Age and gender did not have a significant impact in any models. Table 5 presents the final models for the three types of parenting stress.

**TABLE 5:** Models of psychosis at *t*_0_

	Model of psychosis at *t*_0_ with parenting event stress *t*_−1_		Model of psychosis at *t*_0_ with parenting activity stress *t*_−1_		Model of psychosis at *t*_0_ with parenting social stress *t*_−1_	
	Estimate (95% CI)	*p*‐value	Estimate (95% CI)	*p*‐value	Estimate (95% CI)	*p*‐value
Observation number	−.05 (−.10–.00)	.070	−.04 (−.09–.02)	.185	−.04 (−.07–.00)	.030*
Parenting stress *t*_−1_	.07 (.01–.14)	.022*	.10 (.02–.19)	.014*	.03 (−.01–.06)	.176
Negative affect *t*_−1_	.10 (−.01–.21)	.070	.10 (−.04–.23)	.178	.08 (.01–.16)	.034*
Mean stress	.24 (.03–.46)	.027*	.59 (.31–.87)	<.001*	.14 (−.15–.44)	.339
Mean negative affect	.58 (.40–.77)	<.001*	.39 (.17–.61)	.001*	.57 (.33–.81)	<.001*
Age	−.12 (−.60–.36)	.614	−.14 (−.54–.25)	.459	−.06 (−.57–.45)	.819
Gender (male)	2.43 (−4.88–9.74)	.502	6.00 (−.56–12.56)	.072	4.26 (−4.03–12.55)	.304

*Significance at .05 level.

These data show that stress due to parenting did increase positive psychotic symptoms, and that this effect was mediated by negative affect, meaning Hypothesis 1 was supported.

#### Hypothesis 2: Level‐2 variables improving the models' estimation

The Level‐2 variables (i) social support, (ii) child behaviour, (iii) parenting self‐efficacy and (iv) coping were introduced as covariates into each of the models. None of these had a significant improvement on any models, except child behaviour with the model including parenting social stress *t*_−1_, meaning there was only partial support for Hypothesis 2.

The models were then extended so that each Level‐2 variable, as well as gender, was included as a moderator on the relationship between parenting stress *t*_−1_ and psychosis *t*_0_, in separate models. There was no evidence that gender moderated the relationship between any type of parenting stress *t*_−1_ and psychosis *t*_0_. The other four moderators did have effects in some models. The full models where Level‐2 variables were introduced as covariates, and the models of significant moderations, can be found in Appendix S1.

#### Parenting self‐efficacy

Parenting self‐efficacy was shown to moderate the relationship between all three types of parenting stress *t*_−1_ and psychosis *t*_0_, in that those with lower levels of parenting self‐efficacy had a stronger association between parenting stress *t*_−1_ and psychosis *t*_0_:

- Parenting event stress *t*_−1_: [β(S.E.) = −1.14 (.34), 95% CI [−1.81 to −.48], *p* < .001], parenting event stress *t*_−1_ × parenting self‐efficacy: [β(S.E.) = .34 (.09), 95% CI [.16–.53], *p* < .001]
- Parenting activity stress *t*_−1_: [β(S.E.) = −.62 (.33), 95% CI [−1.26–.02], *p* = .059], parenting activity stress *t*_−1_ × parenting self‐efficacy: [β(S.E.) = .20 (.09), 95% CI [.02–.37], *p* = .030]
- Parenting social stress *t*_−1:_ [β(S.E.) = −.41 (.16), 95% CI [−.71 to −.10], *p* = .009], parenting social stress *t*_−1_ × parenting self‐efficacy: [β(S.E.) = .12 (.04), 95% CI [.03–.20], *p* = .005].

##### Coping

Coping was shown to moderate only the relationship between parenting activity stress *t*_−1_ and psychosis *t*_0_, in that those with lower levels of coping had a stronger association between parenting activity stress *t*_−1_ and psychosis *t*_0_ (parenting activity stress *t*_−1_: [β(S.E.) = −.25 (.11), 95% CI [−.47 to −.03], *p* = .028], parenting activity stress *t*_−1_ × coping: [β(S.E.) = .07 (.02), 95% CI [.03–.11], *p* = .002]).

##### Social support

Social support was shown to moderate only the relationship between parenting social stress *t*_−1_ and psychosis *t*_0_, in that participants with lower social support had a stronger association between parenting social stress *t*_−1_ and psychosis *t*_0_ (parenting social stress *t*_−1_: [β(S.E.) = −.17 (.06), 95% CI [−.30 to −.05], *p* = .008], parenting social stress *t*_−1_ × social support: [β(S.E.) = .06 (.02), 95% CI [.02–.09], *p* = .001]).

##### Child behaviour

Child behaviour was significant in the modelling of parenting social stress *t*_−1_ on psychosis *t*_0_ as a covariate and then as a moderator. The results showed that those who reported a higher total difficulties score experienced less psychosis when all other variables in the model were held equal [β(S.E.) = −.76 (.30), 95% CI [−1.38 to −.15], *p* = .016].

Regarding its moderating effects, those with a higher child behaviour total difficulties score had a weaker association between parenting social stress *t*_−1_ and psychosis *t*_0_ (parenting social stress *t*_−1_: [β(S.E.) = .14 (.04), 95% CI [.06–.21], *p* < .001], parenting social stress *t*_−1_ × child behaviour: [β(S.E.) = −.01 (.00), 95% CI [−.01–.00], *p* = .001]).

#### Hypothesis 3: Stress due to parenting will have a larger impact than general stress

When a type‐of‐stress indicator was included in the modelling of psychosis and stress, there was a significant finding in two of the models. There was no evidence that the association between event stress at *t*_−1_ and psychosis *t*_0_ differed depending on the type of stress (parenting‐related stress or other‐related stress) (see Table 6). However, there was evidence that type‐of‐stress moderated the relationship for both activity stress *t*_−1_ and social stress *t*_−1_, albeit in the opposite direction to what was hypothesized, in that stress related to parenting had a weaker association between stress and psychosis than other types of stress (see Table 6), and therefore Hypothesis 3 was not supported.

**TABLE 6:** Effects of type‐of‐stress indicator in modelling of psychosis *t*_0_

	Model of psychosis at *t*_0_ with event stress *t*_−1_		Model of psychosis at *t*_0_ with activity stress *t*_−1_		Model of psychosis at *t*_0_ with social stress *t*_−1_	
	Estimate (95% CI)	*p*‐value	Estimate (95% CI)	*p*‐value	Estimate (95% CI)	*p*‐value
Observation number	−.05 (−.08 to −.02)	<.001*	−.05 (−.07 to −.02)	.001*	−.06 (−.09 to –.03)	<.001*
Stress *t*_−1_	.02 (−.01–.05)	.151	.07 (.04–.11)	<.001*	.08 (.03–.12)	.001*
Type‐of‐stress (parenting)	−1.01 (−2.49–.48)	.184	.35 (−1.33–2.03)	.686	−.65 (−2.10–.81)	.383
Negative affect *t*_−1_	.05 (.00–.10)	.049*	.04 (−.01–.10)	.100	.06 (.00–.13)	.068
Mean stress	.31 (.12–.49)	.002*	.61 (.38–.83)	<.001*	.63 (.39–.88)	<.001*
Mean negative affect	.59 (.45–.74)	<.001*	.42 (.27–.57)	<.001*	.38 (.22–.55)	<.001*
Age	−.04 (−.46–.38)	.852	.01 (−.33–.35)	.952	−.05 (−.42–.32)	.795
Gender (male)	−.04 (−6.61–6.54)	.991	2.47 (−3.01–7.95)	.366	4.36 (−1.60–10.33)	.146
Stress *t*_−1_ × type‐of‐stress (parenting)	−.03 (−.07–.00)	.075	−.11 (−.15 to −.06)	<.001*	−.06 (−.10 to –.01)	.022*

*Significance at .05 level.

#### Hypothesis 4: The impact of positive psychotic symptoms on parenting stress

The three models of the parenting stress (activity, event and social) at *t*_0_ demonstrated that they were all significantly predicted by psychosis scores at *t*_−1_:

- Parenting event stress: β(S.E.) = .33 (.08), 95% CI [.17–.50], *p* < .001
- Parenting activity stress: β(S.E.) = .44 (.06), 95% CI [.31–.57], *p* < .001
- Parenting social stress: β(S.E.) = .44 (.08), 95% CI [.26–.60], *p* < .001.

Negative affect did not mediate the relationship between psychosis *t*_−1_ and any of the three types of parenting stress at *t*_0_ and therefore negative affect was not included in the modelling of parenting stress (see Table 7). These data show that positive psychotic symptoms did increase stress due to parenting; however, this effect was not mediated by negative affect, meaning Hypothesis 4 was only partially supported.

**TABLE 7:** Mediation effects of negative affect *t*_−1_ on the relationship between psychosis *t*_−1_ and parenting stress *t*_0_

	Parenting event stress		Parenting activity stress		Parenting social stress	
	Estimate (95% CI)	*p*‐value	Estimate (95% CI)	*p*‐value	Estimate (95% CI)	*p*‐value
Average causal mediation effect	−.11 (−.30–.07)	.230	.14 (.01–.29)	.044	.02 (.01–.04)	.350
Average direct effect	.45 (.21–.70)	<.001*	.31 (.12–.50)	.002*	.04 (.01–.07)	<.001*
Total effect	.34 (.18–.50)	<.001*	.46 (.34–.58)	<.001*	.06 (.03–.08)	<.001*
Proportion mediated	−.32 (−1.11–.21)	.230	.31 (.02–.68)	.044*	.36 (.11–.83)	.350

*Significance at .02 level.

Mean levels of psychosis were significant in the modelling of parenting social stress *t*_0_ in that those with higher average levels of psychosis experienced more parenting social stress when all other variables were held equal. Gender and age were significant in the modelling of parenting activity stress *t*_0_, in that men in the sample reported lower levels of parenting stress than women and those who were older experienced more parenting activity stress *t*_0_ (see Table 8).

**TABLE 8:** Models of parenting stress at *t*_0_

	Model of parenting event stress *t*_0_		Model of parenting activity stress *t*_0_		Model of parenting social stress *t*_0_	
	Estimate (95% CI)	*p*‐value	Estimate (95% CI)	*p*‐value	Estimate (95% CI)	*p*‐value
Observation number	.02 (−.08–.12)	.686	.02 (−.08–.12)	.684	−.01 (−.10–.08)	.805
Psychosis *t*_−1_	.34 (.11–.57)	.003*	.29 (.07–.52)	.011*	.17 (−.04–.39)	.109
Mean psychosis	−.02 (−.34–.30)	.918	.23 (−.03–.49)	.085	.53 (.23–.82)	.001*
Age	.38 (−.42–1.18)	.339	.46 (.02–.89)	.041*	−.11 (−.79–.57)	.743
Gender (male)	−9.54 (−22.21–3.14)	.136	−11.28 (−17.96 to −4.60)	.002*	−8.34 (−19.19–2.50)	.127

*Significance at .05 level.

The models were then extended so that all Level‐2 variables were introduced as covariates. None of these had a significant improvement on any models, except child behaviour in the modelling of parenting event stress *t*_0_. Then each Level‐2 variable was included as a moderator on the relationship between psychosis *t*_−1_ and parenting stress *t*_0_ in separate models. There was no evidence that social support moderated the relationship between psychosis *t*_−1_ and any type of parenting stress *t*_0_. Appendix S1 contains the full models with Level‐2 variables as covariates and the significant moderations.

##### Parenting self‐efficacy

Parenting self‐efficacy had a significant effect as a moderator, in that those with higher parenting self‐efficacy had a weaker association between psychosis *t*_−1_ and parenting social stress *t*_0_ (psychosis *t*_−1_: [β(S.E.) = 1.76 (.57), 95% CI [.61–2.91], *p* = .003], psychosis *t*_−1_ × parenting self‐efficacy: [β(S.E.) = −.41 (.15), 95% CI [−.70 to −.12], *p* = .007]).

##### Coping

When coping was introduced as a moderator, those with higher levels of coping had a weaker association between psychosis *t*_−1_ and parenting event stress *t*_0_ (psychosis *t*_−1_: [β(S.E.) = −.22 (.21), 95% CI [−.63–.20], *p* = .305], psychosis *t*_−1_ × coping: [β(S.E.) = .11 (.04), 95% CI [.04–.19], *p* = .005]).

##### Child behaviour

Child behaviour had significant effects as a covariate in the modelling of parenting event stress *t*_0_ in that those with a higher child total difficulties score experienced less parenting stress when all other covariates were held equal [β(S.E.) = −.96 (.38), 95% CI [−1.73 to −.19], *p* = .016].

Furthermore, while coping did not significantly moderate psychosis *t*_−1_ in the modelling of parenting event stress *t*_0_, it did moderate psychosis *t*_−1_ in the modelling of parenting social stress *t*_0_, in that those with higher total difficulties score had a weaker association between psychosis *t*_−1_ and parenting social stress *t*_0_ (psychosis *t*_−1_: [β(S.E.) = −.03 (.15), 95% CI [−.34–.27], *p* = .828]), (psychosis *t*_−1_ × child behaviour: [β(S.E.) = .02 (.01), 95% CI [.00–.04], *p* = .030]).

##### Gender

Gender had a significant effect as a moderator in both the modelling of parenting activity stress *t*_0_ and parenting social stress *t*_0_, in that men had a weaker association between psychosis *t*_−1_ and parenting stress *t*_0_.

- Parenting activity stress *t*_0_: (psychosis *t*_−1_: [β(S.E.) = .43 (.13), 95% CI [.17–.69], *p* = .001], psychosis *t*_−1_ × gender (male): [β(S.E.) = −.36 (.12), 95% CI [−.60 to −.12], *p* = .004])
- Parenting social stress *t*_0_: (psychosis *t*_−1_: [β(S.E.) = .29 (.13), 95% CI [.04–.55], *p* = .023], psychosis *t*_−1_ × gender (male): [β(S.E.) = −.53 (.18), 95% CI [−.90 to −.16], *p* = .006]).

#### Sensitivity analysis of missing data

Missing data were imputed for the Level‐1 variables using the ‘mice’ package in R. Missing data were imputed using a two‐level normal model, and forty imputations were performed to create ten different datasets which were pooled together for analyses. Diagnostic plots for the multiple imputation are displayed in Appendix S2.

Each model was rerun with the imputed data, and the results from each imputation were pooled to obtain the model results. The estimates and *p*‐values of these models were compared to those in the original models. Child behaviour only remained significant as a covariate, and not as a moderator, in the modelling of psychosis *t*_0_ with parenting social stress *t*_−1_. The effect of child behaviour as a covariate in the modelling of parenting event stress *t*_0_, and its moderating effects on psychosis *t*_−1_ in the modelling of parenting social stress *t*_0_ disappeared. Age and gender were no longer significant as covariates in the modelling of parenting activity stress *t*_0_.

None of the originally significant moderations of other Level‐2 variables on parenting stress *t*_−1_ when modelling psychosis *t*_0_ remained significant, except for the moderation of coping on parenting activity stress *t*_−1_. Similarly, when modelling parenting stress *t*_0_, none of the Level‐2 moderators remained significant, except for the moderation of parenting self‐efficacy on psychosis *t*_−1_ when modelling parenting social stress *t*_0_.

When checking the effects of the type‐of‐stress moderation (parenting‐related or other‐related), only one interaction remained significant, which was the modelling of psychosis *t*_0_ containing parenting activity stress *t*_−1_.

The models from the imputed data can be seen in Appendix S2.

#### Post hoc exploratory analysis

Subsequent to the in‐principle‐acceptance of this study's protocol, Klippel et al. (2021) published the results of their study looking at both the temporal and cross‐sectional relationship between stress and psychosis, and only found evidence for a cross‐sectional relationship. The design in this study assumed a temporal relationship without investigating a cross‐sectional one. Specifically, the original models modelled current psychosis using lagged parenting stress, which was found to play a significant role (and vice‐versa). These models did not account for the association between current parenting stress and current psychosis, and therefore could not demonstrate whether there is indeed a temporal relationship or whether the relationship is solely cross‐sectional. The models also did not account for auto‐correlation, which denotes how much current psychosis is associated with lagged psychosis.

Therefore, we decided to model for a cross‐sectional relationship post hoc as well as looking at auto‐correlation of the outcome variable. In order to model these two additional effects, parenting stress *t*_0_ and psychosis *t*_−1_ were included in the modelling of psychosis *t*_0_. To model the opposite direction, psychosis *t*_0_ and parenting stress *t*_−1_ were included in the modelling of parenting stress *t*_0_.

Both of these additional covariates were found to be highly significant in all models, and the temporal relationship between lagged parenting stress and current psychosis (and vice‐versa) disappeared. Therefore, it seems that the relationship is mainly cross‐sectional. This exploratory analysis can be found in Appendix S3.

## DISCUSSION

### Key findings

This study has provided further evidence for the relationship between psychotic symptoms, stress and low mood, in a novel sample of parents with a diagnosed psychotic disorder. There is a large literature demonstrating the relationship between positive psychotic symptoms and stress in ESM studies (e.g. Palmier‐Claus et al., 2012; van der Steen et al., 2017), and in non‐intensive longitudinal designs (DeVylder et al., 2013; Tessner et al., 2011). This study has shown that this relationship also holds true for stress due to parenting, in that more stressful parenting experiences predict a rise in psychotic symptoms, which supports Hypothesis 1.

It also further confirmed the importance of negative affect as a mediator of the relationship between stress and psychotic symptoms. Negative affect was found to mediate all three types of parenting stress: social stress (stress in the company of children), event stress (stress from a recent parenting event) and activity stress (stress from a current parenting activity). In some models, when negative affect was included with stress in the modelling of psychosis, the direct impact of stress became non‐significant. Negative affect, or low mood, has been shown to be one of the key mechanisms of the stress‐sensitivity hypothesis (Betz et al., 2020; Klippel et al., 2017, 2018), also known as the affective pathway to psychosis (Myin‐Germeys & van Os, 2007). Negative affect seemingly increases aberrant salience, whereby one attaches meaning to ambiguous events (So et al., 2018). Introducing participants' mean levels of negative affect after stressful events, a measure of stress sensitivity (Kramer et al., 2014), improved models' estimation showing the impact of participants' general levels of low mood on psychotic symptoms.

Psychosis can be frightening and unfamiliar, especially for those for whom these symptoms are relatively new, as was the case for some participants in this sample. Whilst much research has investigated the impact on stress of psychosis, less attention has been paid to the reverse relationship (Klippel et al., 2021). This study demonstrated that the relationship between stress from parenting and psychosis is not unidirectional; although parenting stress predicted psychotic symptoms, levels of psychosis were also shown to predict parenting stress, supporting Hypothesis 4. However, this reverse association did not seem to be as strong. Once other covariates were introduced into the modelling of parenting stress, the effects of psychosis became non‐significant in some models. Furthermore, negative affect did not play an important role as it did not mediate the pathway from psychosis to parenting stress. These findings suggest that there are further complexities that exist when modelling this reverse pathway, and more attention must be paid to it when investigating the relationship between stress and psychosis.

This study assumed a temporal relationship between parenting stress and psychosis, and vice‐versa. However, research looking at both the cross‐sectional and temporal relationship that was published after this study began (Klippel et al., 2021) did not find evidence for any temporal priority. The authors instead concluded that the temporal relationships between these factors may be more complex than can be observed by time lagging of a couple of hours. The exploratory analyses in this study also confirmed this; once cross‐sectional variables were introduced into the models, the effects of their lagged equivalents disappeared.

We did not confirm Hypothesis 3, that parenting stress would have a larger impact on psychotic symptoms than other types of stress. Indeed, it seemed there was weak evidence for the opposite effect whereby non‐parenting‐related stress had a larger impact on the relationship. It may be the case that the design of this study made it difficult to disentangle the effects of stress due to parenting and other stress due to the indirect ways that parenting activities can also impact on daily lives. We can still conclude nevertheless, that stress is important in predicting psychotic symptoms in parents with psychosis. It may have been beneficial to compare these participants to a group of non‐parent participants to fully unpack the effects of parenting stress.

Finally, there was little evidence for Hypothesis 2, that including measures of (i) child behaviour, (ii) coping, (iii) parenting self‐efficacy, (iv) social support or (v) gender as covariates or moderators of the relationship between stress and psychosis improved the models' estimations. Some effects were found in the initial modelling, but these did not remain significant after conducting a sensitivity analysis using multiple imputation of missing data. Coping and parenting self‐efficacy both remained significant as moderators in separate models after the sensitivity analysis; however, this was the case for only one type of parenting stress in both instances. It may be the case that this lack of effect is due to a high proportion of variance already being accounted for by the Level‐1 (ESM) variables. Investigating these factors as time‐varying variables (e.g. ‘right now, I am coping well’) rather than time‐invariant (Level‐2) factors may have given more insight into their relationship with stress and psychosis.

### Strengths and limitations

Current research on parenting with severe mental illnesses such as psychosis is primarily formed of qualitative work and non‐intensive outcome measures. To our knowledge, this is the first study to use experience sampling methodology to investigate daily fluctuations of mood, stress and psychosis within a sample of parents with a diagnosed psychotic disorder. Furthermore, it adds to the stress‐sensitivity literature on psychosis by presenting further confirmatory evidence of the bidirectional relationship between stress and psychosis, and the importance of negative affect as a mediator.

One limitation of the study is the way psychosis was measured. Whereas the full set of ESM variables for negative affect and stress was used in other ESM studies (e.g. Palmier‐Claus et al., 2012; Reininghaus et al., 2016; van der Steen et al., 2017), the variables for psychosis were created by looking at examples from other studies and from guidance from parents with psychosis. While ESM variables are still not as well‐validated as other more traditional psychometric measures, efforts are underway to improve the validity and reliability of ESM measures (https://osf.io/kg376/). Another potential limitation is that many of participants in the sample were married and employed, which is more likely with parents with psychosis (Radley et al., 2022), but not representative of the majority of individuals with psychosis (Perälä et al., 2008). This may limit the generalizability of these findings. A final limitation is that introducing lagged variables does not fully account for the complexities of modelling reverse causality, as it is not clear how much of the reverse causality is explained by the lagged variables.

### Implications for practice and future research

Future research should aim to explore further whether there is any impact of parenting stress on psychotic symptoms over and above that of general stress. Parents with psychosis should be compared to non‐parents with psychosis to investigate if any differences exist in the relationship between stress and psychosis in these two groups.

Additionally, it is clear from the literature that the temporal relationship between stress, psychosis and negative affect is complex (Klippel et al., 2021). The exploratory analysis in this study found no evidence for a temporal relationship between parenting stress and psychosis once the cross‐sectional relationship was included in the modelling. More work should be done to disentangle these effects using a more rapid sampling frame or techniques such as network analysis.

Parenting is stressful, and clinicians should work to consider clients' parenting status and the role these daily stressors have in exacerbating psychotic symptoms. Importantly, clinicians should be mindful that it is clients' perceptions of their daily stressors and the significance attributed to them which forms the affective pathway to psychosis.

## CONCLUSIONS

We hypothesized (1) parenting stress would increase psychotic symptoms, mediated by negative affect, (2) certain psychosocial factors would improve models' estimation, (3) stress due to parenting would have a stronger effect than general stress in this relationship and (4) the opposite relationship: psychotic symptoms would increase parenting stress, mediated by negative affect. We were able to confirm Hypotheses 1 and 4 and in doing so, provide more evidence for the bidirectional nature of stress and psychosis in the context of parenting. There was weak evidence for Hypothesis 2, that any of the time invariant factors – gender, coping, child behaviour, parenting self‐efficacy, coping – affected the relationship between stress and psychosis. Finally, we found no evidence for Hypothesis 3, that parenting stress was more important than general stress in the modelling of stress and psychosis. Further research should explore the role of parenting stress in psychosis by comparing parents and non‐parents with psychosis, and use novel designs to disentangle the directionality of the relationship between stress and psychosis.

## AUTHOR CONTRIBUTIONS

**Jessica Radley:** Conceptualization; formal analysis; investigation; methodology; project administration; writing – original draft. **Jane Barlow:** Conceptualization; supervision; writing – review and editing. **Louise Johns:** Conceptualization; methodology; supervision; writing – review and editing.

## FUNDING INFORMATION

JR is a DPhil student and is funded by Mental Health Research UK. No funding was received for conducting this study.

## CONFLICT OF INTEREST

The authors declare that they have no conflict of interest.

## AVAILABILITY OF DATA AND MATERIAL

All data used in this analysis can be found here https://osf.io/wj3cv/.

## CODE AVAILABILITY

The R code used in this analysis is available here https://github.com/JessRadley/ESM‐parents‐with‐psychosis.

## ETHICS APPROVAL

This study was given ethical approval by London Bridge Research Ethics Committee'.

## STAGE 1 PROTOCOL

https://osf.io/wj3cv/ (DOI 10.17605/OSF.IO/WJ3CV).

## CHANGES FROM PROTOCOL

Maximum likelihood estimation (ML) was used instead of restricted maximum likelihood (REML) due to learning obtained by the authors at a statistics course after this manuscript received in‐principle‐acceptance. ML can achieve model stability in cases where there are at least 30 clusters (Hox & McNeish, 2020), and therefore is the appropriate choice for this study's design.

## Supporting information


Appendix S1

Appendix S2

Appendix S3

## Data Availability

http://doi.org/10.17605/OSF.IO/WJ3CV.
