# Supplementary material for: Parenting and psychosis: An experience sampling methodology study investigating the inter‐relationship between stress from parenting and positive psychotic symptoms
Source: Br J Clin Psychol. 2022 Aug 8;61(4):1236–58. doi: 10.1111/bjc.12389 (PMC9804428; doi:10.1111/bjc.12389)
Supplement: Supplementary file 1 — Appendix S1 [file BJC-61-1236-s003.docx]

Table 1 - Modelling of psychosis at t_0_ with Level-2 variables included as covariates

|  | Model of psychosis at t_0_ with parenting event stress t_-1_ | | Model of psychosis at t_0_ with parenting activity stress t_-1_ | | Model of psychosis at t_0_ with parenting social stress t_-1_ | |
| --- | --- | --- | --- | --- | --- | --- |
|  | Estimate (95% CI) | p-value | Estimate (95% CI) | p-value | Estimate (95% CI) | p-value |
| Observation number | -0.03 (-0.09-0.02) | 0.233 | -0.03 (-0.08-0.03) | 0.354 | -0.02 (-0.06-0.01) | 0.172 |
| Parenting stress t_-1_ | 0.09 (0.02-0.15) | **0.011*** | 0.09 (0.01-0.17) | **0.036*** | 0.03 (-0.01-0.06) | 0.129 |
| Negative affect t_-1_ | 0.09 (-0.02-0.20) | 0.106 | 0.08 (-0.06-0.22) | 0.245 | 0.07 (-0.01-0.15) | 0.071 |
| Mean stress | 0.29 (0.05-0.53) | **0.021*** | 0.63 (0.35-0.91) | **<0.001*** | 0.20 (-0.09-0.50) | 0.171 |
| Mean negative affect | 0.69 (0.43-0.95) | **<0.001*** | 0.52 (0.26-0.77) | **<0.001*** | 0.70 (0.42-0.98) | **<0.001*** |
| Age | -0.12 (-0.62-0.38) | 0.619 | -0.13 (-0.54-0.29) | 0.538 | 0.07 (-0.48-0.61) | 0.801 |
| Gender (male) | 1.26 (-6.71-9.23) | 0.750 | 5.50 (-1.87-12.87) | 0.138 | 2.62 (-5.90-11.14) | 0.536 |
| Social support (MOS Survey) | 0.49 (-3.24-4.21 | 0.791 | 1.25 (-2.06-4.56) | 0.447 | -0.66 (-4.80-3.49) | 0.749 |
| Child behaviour (SDQ) | -0.46 (-1.05-0.13) | 0.125 | -0.34 (-0.83-0.16) | 0.175 | -0.76 (-1.38- -0.15) | **0.016*** |
| Coping (CSES) | 1.03 (-1.52-3.59) | 0.413 | 1.62 (-0.51-3.76) | 0.131 | 1.06 (-1.65-3.78) | 0.431 |
| Parenting self-efficacy (MaaP) | 0.71 (-10.71-12.13) | 0.900 | -5.27 (-14.41-3.87) | 0.248 | -4.07 (-16.14-8.00) | 0.497 |

Table 2 - Modelling psychosis t_0_ with significant Level-2 significant moderator and parenting event stress t_-1_

|  | Model of psychosis t_0_ with parenting self-efficacy x parenting event stress t_-1_ | |
| --- | --- | --- |
|  | Estimate (95% CI) | p-value |
| Observation number | -0.03 (-0.09-0.02) | 0.189 |
| Parenting event stress t_-1_ | -1.14 (-1.81- -0.48) | **0.001*** |
| Negative affect t_-1_ | 0.09 (-0.02-0.19) | 0.099 |
| Mean event stress | 0.24 (-0.02-0.49) | 0.068 |
| Mean negative affect | 0.74 (0.47-1.01) | **<0.001*** |
| Age | -0.02 (-0.55-0.51) | 0.939 |
| Gender (male) | 1.40 (-7.06-9.86) | 0.738 |
| Social support (MOS Survey) | 0.51 (-3.46-4.49) | 0.794 |
| Child behaviour (SDQ) | -0.57 (-1.20-0.07) | 0.078 |
| Coping (CSES) | 1.26 (-1.49-4.01) | 0.354 |
| Parenting self-efficacy (MaaP) | -7.18 (-19.89-5.53) | 0.260 |
| Parenting event stress t_-1_ x Parenting self-efficacy (MaaP) | 0.34 (0.16-0.53) | **<0.001*** |

Table 3 - Modelling psychosis t_0_ with significant Level-2 significant moderators and parenting activity stress t_-1_

|  | Model of psychosis t_0_ with parenting activity stress t_-1_ x coping | | Model of psychosis t_0_ with parenting activity stress t_-1_ x parenting self-efficacy | |
| --- | --- | --- | --- | --- |
|  | Estimate (95% CI) | p-value | Estimate (95% CI) | p-value |
| Observation number | -0.03 (-0.09-0.02) | 0.233 | -0.03 (-0.08-0.03) | 0.289 |
| Parenting activity stress t_-1_ | -0.25 (-0.47- -0.03) | **0.028*** | -0.62 (-1.26-0.02) | 0.059 |
| Negative affect t_-1_ | 0.10 (-0.04-0.23) | 0.151 | 0.08 (-0.05-0.22) | 0.218 |
| Mean activity stress | 0.58 (0.27-0.88) | **<0.001*** | 0.61 (0.31-0.90) | **<0.001*** |
| Mean negative affect | 0.56 (0.30-0.83) | **<0.001*** | 0.56 (0.29-0.82) | **<0.001*** |
| Age | -0.10 (-0.54-0.35) | 0.662 | -0.09 (-0.52-0.34) | 0.685 |
| Gender (male) | 4.49 (-3.40-12.38) | 0.255 | 4.59 (-3.09-12.27) | 0.232 |
| Social support (MOS Survey) | 1.29 (-2.25-4.82) | 0.463 | 1.37 (-2.07-4.80) | 0.423 |
| Child behaviour (SDQ) | -0.47 (-1.00-0.07) | 0.085 | -0.42 (-0.94-0.10) | 0.111 |
| Coping (CSES) | 0.50 (-1.89-2.89) | 0.671 | 1.74 (-0.49-3.97) | 0.122 |
| Parenting self-efficacy (MaaP) | -5.75 (-15.52-4.02) | 0.239 | -8.95 (-18.94-1.03) | 0.077 |
| Parenting activity stress t_-1_ x Coping (CSES) | 0.07 (0.03-0.11) | **0.002*** |  |  |
| Parenting activity stress t_-1_ x Parenting self-efficacy (MaaP) |  |  | 0.20 (0.02-0.37) | **0.030*** |

Table 4 - Modelling psychosis t_0_ with significant Level-2 significant moderators and parenting social stress t_-1_

|  | Model of psychosis t_0_ with parenting social stress t_-1_ x social support | | Model of psychosis t_0_ with parenting social stress t_-1_ x child behaviour | | Model of psychosis t_0_ with parenting social stress t_-1_ x parenting self-efficacy | |
| --- | --- | --- | --- | --- | --- | --- |
|  | Estimate (95% CI) | p-value | Estimate (95% CI) | p-value | Estimate (95% CI) | p-value |
| Observation number | -0.03 (-0.06-0.00) | 0.082 | -0.03 (-0.06-0.01) | 0.100 | -0.03 (-0.06-0.00) | 0.087 |
| Parenting social stress t_-1_ | -0.17 (-0.30- -0.05) | **0.008*** | 0.14 (0.06-0.21) | **<0.001*** | -0.41 (-0.71- -0.10) | **0.009*** |
| Negative affect t_-1_ | 0.06 (-0.01-0.14) | 0.104 | 0.05 (-0.02-0.13) | 0.167 | 0.07 (0.00-0.15) | 0.054 |
| Mean social stress | 0.23 (-0.07-0.54) | 0.132 | 0.25 (-0.02-0.53) | 0.072 | 0.26 (-0.03-0.55) | 0.081 |
| Mean negative affect | 0.72 (0.43-1.00) | **<0.001*** | 0.73 (0.46-0.99) | **<0.001*** | 0.72 (0.44-0.99) | **<0.001*** |
| Age | 0.11 (-0.45-0.67) | 0.689 | 0.06 (-0.44-0.57) | 0.797 | 0.11 (-0.41-0.64) | 0.666 |
| Gender (male) | 2.42 (-6.32-11.16) | 0.578 | 3.15 (-4.78-11.09) | 0.425 | 2.54 (-5.70-10.79) | 0.535 |
| Social support (MOS Survey) | -1.40 (-5.67-2.88) | 0.512 | -0.24 (-4.10-3.62) | 0.899 | -0.45 (-4.47-3.56) | 0.819 |
| Child behaviour (SDQ) | -0.79 (-1.42- -0.16) | **0.016*** | -0.54 (-1.12-0.04) | 0.069 | -0.76 (-1.35- -0.16) | **0.014*** |
| Coping (CSES) | 1.14 (-1.65-3.93) | 0.410 | 1.11 (-1.41-3.63) | 0.374 | 1.12 (-1.51-3.74) | 0.393 |
| Parenting self-efficacy (MaaP) | -4.06 (-16.45-8.33) | 0.509 | -4.56 (-15.78-6.67) | 0.414 | -6.16 (-17.92-5.60) | 0.294 |
| Parenting social stress t_-1_ x social support (MOS survey) | 0.06 (0.02-0.09) | **0.001*** |  |  |  |  |
| Parenting social stress t_-1_ x child behaviour (SDQ) |  |  | -0.01 (-0.01-0.00) | **0.001*** |  |  |
| Parenting social stress t_-1_ x Parenting self-efficacy (MaaP) |  |  |  |  | **0.12 (0.03-0.20)** | **0.005*** |

Table 5 - Modelling of parenting stress at t_0_ with Level 2 variables included as covariates

|  | Model of parenting event stress t_0_ | | Model of parenting activity stress t_0_ | | Model of parenting social stress t_0_ | |
| --- | --- | --- | --- | --- | --- | --- |
|  | Estimate (95% CI) | p-value | Estimate (95% CI) | p-value | Estimate (95% CI) | p-value |
| Observation number | 0.06 (-0.04-0.15) | 0.252 | 0.04 (-0.07-0.14) | 0.486 | -0.01 (-0.11-0.08) | 0.800 |
| Psychosis t_-1_ | 0.28 (0.05-0.52) | **0.017*** | 0.28 (0.04-0.53) | **0.021*** | 0.18 (-0.06-0.42) | 0.143 |
| Mean psychosis | 0.12 (-0.25-0.50) | 0.513 | 0.10 (-0.19-0.39) | 0.510 | 0.23 (-0.15-0.61) | 0.232 |
| Age | 0.38 (-0.33-1.09) | 0.286 | 0.36 (-0.06-0.79) | 0.088 | -0.40 (-1.08-0.28) | 0.239 |
| Gender (male) | -13.93 (-26.46- -1.39) | **0.030*** | -11.69 (-18.72- -4.66) | **0.002*** | -5.60 (-16.75-5.55) | 0.314 |
| Social support (MOS Survey) | -1.06 (-6.63-4.51) | 0.701 | 0.31 (-3.15-3.77) | 0.858 | 2.82 (-2.42-8.07) | 0.280 |
| Child behaviour (SDQ) | -0.96 (-1.73- -0.19) | **0.016*** | 0.16 (-0.33-0.64) | 0.510 | 0.65 (-0.07-1.37) | 0.074 |
| Coping (CSES) | 0.76 (-2.49-4.02) | 0.635 | -1.46 (-3.38-0.47) | 0.131 | -1.94 (-5.06-1.18) | 0.212 |
| Parenting self-efficacy (MaaP) | -14.97 (-31.60-1.67) | 0.076 | -7.10 (-16.63-2.43) | 0.138 | -6.03 (-21.67-9.61) | 0.438 |

Table 6 - Modelling psychosis t_-1_ on parenting event stress t_0_ with significant Level-2 significant moderators

|  | Model of parenting event stress t_0_ with psychosis t_-1_ x coping | |
| --- | --- | --- |
|  | Estimate (95% CI) | p-value |
| Observation number | 0.05 (-0.05-0.14) | 0.323 |
| Psychosis t_-1_ | -0.22 (-0.63-0.20) | 0.305 |
| Mean psychosis | 0.21 (-0.15-0.57) | 0.240 |
| Age | 0.32 (-0.32-0.97) | 0.312 |
| Gender (male) | -13.39 (-24.93- -1.86) | **0.024*** |
| Social support (MOS Survey) | 0.27 (-4.84-5.38) | 0.915 |
| Child behaviour (SDQ) | -0.97 (-1.67- -0.28) | **0.008*** |
| Coping (CSES) | -1.97 (-5.42-1.47) | 0.253 |
| Parenting self-efficacy (MaaP) | -15.19 (-30.33- -0.06) | **0.049*** |
| Psychosis t_-1_ x Coping (CSES) | 0.11 (0.04-0.19) | **0.005*** |

Table 6 - Modelling psychosis t_-1_ on parenting activity stress t_0_ with significant Level-2 significant moderators

|  | Model of parenting activity stress t_0_ with psychosis t_-1_ x gender | |
| --- | --- | --- |
|  | Estimate (95% CI) | p-value |
| Observation number | 0.04 (-0.06-0.14) | 0.472 |
| Psychosis t_-1_ | 0.43 (0.17-0.69) | **0.001*** |
| Mean psychosis | 0.12 (-0.16-0.40) | 0.393 |
| Age | 0.37 (0.00-0.74) | **0.050*** |
| Gender (male) | -4.07 (-12.42-4.29) | 0.334 |
| Social support (MOS Survey) | 0.31 (-2.75-3.37) | 0.837 |
| Child behaviour (SDQ) | 0.03 (-0.40-0.45) | 0.893 |
| Coping (CSES) | -1.63 (-3.29-0.04) | 0.055 |
| Parenting self-efficacy (MaaP) | -1.40 (-10.70-7.91) | 0.763 |
| Psychosis t_-1_ x Gender (male) | -0.36 (-0.60- -0.12) | **0.004*** |

Table 7 - Modelling psychosis t_-1_ on parenting social stress t_0_ with significant Level-2 significant moderators

|  | Model of parenting social stress t_0_ with psychosis t_-1_ x child behaviour | | Model of parenting social stress t_0_ with psychosis t_-1_ x parenting self-efficacy | | Model of parenting social stress t_0_ with psychosis t_-1_ x gender | |
| --- | --- | --- | --- | --- | --- | --- |
|  | Estimate (95% CI) | p-value | Estimate (95% CI) | p-value | Estimate (95% CI) | p-value |
| Observation number | -0.02 (-0.11-0.08) | 0.705 | -0.02 (-0.11-0.08) | 0.736 | -0.02 (-0.11-0.08) | 0.728 |
| Psychosis t_-1_ | -0.03 (-0.34-0.27) | 0.828 | 1.76 (0.61-2.91) | **0.003*** | 0.29 (0.04-0.55) | **0.023*** |
| Mean psychosis | 0.24 (-0.12-0.60) | 0.194 | 0.15 (-0.21-0.51) | 0.401 | 0.31 (-0.05-0.67) | 0.086 |
| Age | -0.22 (-0.86-0.43) | 0.502 | -0.32 (-0.92-0.28) | 0.290 | -0.41 (-1.02-0.20) | 0.183 |
| Gender (male) | -5.77 (-16.08-4.53) | 0.262 | -3.58 (-13.66-6.49) | 0.474 | 5.38 (-7.36-18.12) | 0.400 |
| Social support (MOS Survey) | 2.03 (-2.86-6.92) | 0.403 | 1.63 (-3.11-6.37) | 0.489 | 2.78 (-1.95-7.52) | 0.240 |
| Child behaviour (SDQ) | -0.04 (-0.95-0.87) | 0.923 | 0.54 (-0.10-1.18) | 0.092 | 0.58 (-0.07-1.23) | 0.078 |
| Coping (CSES) | -1.90 (-4.77-0.96) | 0.183 | -1.99 (-4.75-0.76) | 0.148 | -2.08 (-4.88-0.73) | 0.140 |
| Parenting self-efficacy (MaaP) | -1.99 (-16.94-12.97) | 0.789 | 8.08 (-9.23-25.39) | 0.351 | 0.84 (-14.15-15.83) | 0.910 |
| Psychosis t_-1_ x Child behaviour (SDQ) | 0.02 (0.00-0.04) | **0.030*** |  |  |  |  |
| Psychosis t_-1_ x Parenting Self-Efficacy (MaaP) |  |  | -0.41 (-0.70- -0.12) | **0.007*** |  |  |
| Psychosis t_-1_ x Gender (male) |  |  |  |  | -0.53 (-0.90- -0.16) | **0.006*** |
