# Supplementary material for: Parenting and psychosis: An experience sampling methodology study investigating the inter‐relationship between stress from parenting and positive psychotic symptoms
Source: Br J Clin Psychol. 2022 Aug 8;61(4):1236–58. doi: 10.1111/bjc.12389 (PMC9804428; doi:10.1111/bjc.12389)
Supplement: Supplementary file 2 — Appendix S2 [file BJC-61-1236-s002.docx]

# Sensitivity analysis – models with missing data imputed using multiple imputation


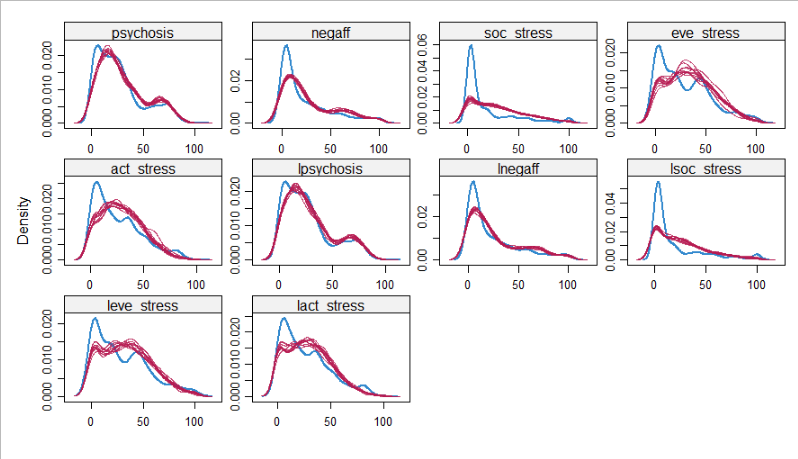


Figure 1 - Density plots of observed data (blue) and imputed data (red) for Level-1 variables

## Modelling psychosis at t0

Table 1 - Models of psychosis at t_0_ with multiple imputation for missing data

|  | Model of psychosis at t_0_ with event stress t_-1_ | | Model of psychosis at t_0_ with activity stress t_-1_ | | Model of psychosis at t_0_ with social stress t_-1_ | |
| --- | --- | --- | --- | --- | --- | --- |
|  | Estimate (95% CI) | p-value | Estimate (95% CI) | p-value | Estimate (95% CI) | p-value |
| Observation number | -0.03 (-0.05- -0.01) | **0.019*** | -0.03 (-0.05- -0.01) | **0.017*** | -0.03 (-0.05-0.00) | **0.024*** |
| Stress t_-1_ | 0.01 (-0.02-0.04) | 0.542 | 0.04 (0.01-0.08) | **0.027*** | 0.05 (0.01-0.09) | **0.013*** |
| Negative affect t_-1_ | 0.05 (0.00-0.10) | 0.051 | 0.04 (-0.01-0.08) | 0.111 | 0.04 (0.00-0.09) | 0.058 |
| Mean stress | 0.29 (0.11-0.47) | **0.001*** | 0.57 (0.35-0.79) | **<0.001*** | 0.16 (-0.10-0.42) | 0.218 |
| Mean negative affect | 0.59 (0.45-0.73) | **<0.001*** | 0.44 (0.29-0.59) | **<0.001*** | 0.60 (0.39-0.81) | **<0.001*** |
| Age | -0.05 (-0.46-0.35) | 0.800 | 0.01 (-0.32-0.35) | 0.934 | 0.02 (-0.43-0.46) | 0.945 |
| Gender (male) | 0.59 (-5.76-6.95) | 0.855 | 2.38 (-3.01-7.76) | 0.387 | 1.12 (-6.02-8.26) | 0.759 |

Table 2 - Models of psychosis at t_0_ with Level-2 variables included as covariates with multiple imputation for missing data

|  | Model of psychosis at t_0_ with event stress t_-1_ | | Model of psychosis at t_0_ with activity stress t_-1_ | | Model of psychosis at t_0_ with social stress t_-1_ | |
| --- | --- | --- | --- | --- | --- | --- |
|  | Estimate (95% CI) | p-value | Estimate (95% CI) | p-value | Estimate (95% CI) | p-value |
| Observation number | -0.03 (-0.05- -0.01) | **0.019*** | -0.03 (-0.05-0.00) | **0.017*** | -0.03 (-0.05-0.00) | **0.024*** |
| Stress t_-1_ | 0.01 (-0.02-0.04) | 0.542 | 0.04 (0.01-0.08) | **0.027*** | 0.05 (0.01-0.09) | **0.012*** |
| Negative affect t_-1_ | 0.05 (0.00-0.10) | 0.052 | 0.04 (-0.01-0.08) | 0.112 | 0.04 (0.00-0.09) | 0.058 |
| Mean stress | 0.30 (0.11-0.48) | **0.002*** | 0.53 (0.29-0.76) | **<0.001*** | 0.16 (-0.09-0.40) | 0.213 |
| Mean negative affect | 0.66 (0.49-0.84) | **<0.001*** | 0.44 (0.25-0.63) | **<0.001*** | 0.62 (0.39-0.85) | **<0.001*** |
| Age | -0.06 (-0.47-0.35) | 0.780 | -0.06 (-0.41-0.30) | 0.759 | 0.03 (-0.43-0.48) | 0.913 |
| Gender (male) | -0.67 (-6.82-5.49) | 0.832 | 1.89 (-3.68-7.47) | 0.506 | -0.72 (-7.54-6.10) | 0.836 |
| Social support (MOS Survey) | 0.25 (-2.81-3.32) | 0.871 | 0.30 (-2.39-3.00) | 0.825 | -1.02 (-4.36-2.33) | 0.550 |
| Child behaviour (SDQ) | -0.53 (-0.96- -0.10) | **0.016*** | -0.25 (-0.66-0.16) | 0.235 | -0.59 (-1.07- -0.11) | **0.016*** |
| Coping (CSES) | 0.86 (-1.06-2.78) | 0.379 | 0.07 (-1.66-1.81) | 0.933 | -0.03 (-2.10-2.03) | 0.975 |
| Parenting self-efficacy (MaaP) | -3.83 (-11.20-4.35) | 0.359 | -5.54 (-12.76-1.69) | 0.133 | -4.77 (-13.58-4.05) | 0.289 |

Table 3 - Model of psychosis t_0_ with Level-2 significant moderators and event stress t_-1_ with multiple imputation for missing data

|  | Model of psychosis at t_0_ with event stress t_-1_ x parenting self-efficacy | |
| --- | --- | --- |
|  | Estimate (95% CI) | p-value |
| Observation number | -0.03 (-0.05- -0.01) | **0.019*** |
| Event stress t_-1_ | -0.04 (-0.19-0.11) | 0.601 |
| Negative affect t_-1_ | 0.05 (0.00-0.10) | 0.062 |
| Mean event stress | 0.30 (0.11-0.48) | **0.002*** |
| Mean negative affect | 0.66 (0.49-0.84) | **<0.001*** |
| Age | -0.06 (-0.46-0.35) | 0.790 |
| Gender (male) | -0.68 (-6.79-5.44) | 0.828 |
| Social support (MOS Survey) | 0.27 (-2.77-3.32) | 0.862 |
| Child behaviour (SDQ) | -0.52 (-0.95- -0.10) | **0.016*** |
| Coping (CSES) | 0.87 (-1.04-2.77) | 0.373 |
| Parenting self-efficacy (MaaP) | -4.22 (-12.39-3.95) | 0.311 |
| Event stress t_-1_ x Parenting self-efficacy (MaaP) | 0.01 (-0.03-0.06) | 0.522 |

Table 4 - Models of psychosis t_0_ with Level-2 significant moderators and activity stress t_-1_ with multiple imputation for missing data

|  | Model of psychosis at t_0_ with activity stress t_-1_ x coping | | Model of psychosis at t_0_ with activity stress t_-1_ x parenting self-efficacy | |
| --- | --- | --- | --- | --- |
|  | Estimate (95% CI) | p-value | Estimate (95% CI) | p-value |
| Observation number | -0.03 (-0.05- -0.01) | **0.015*** | -0.03 (-0.05- -0.01) | **0.017*** |
| Activity stress t_-1_ | -0.04 (-0.11-0.03) | 0.277 | 0.12 (-0.08-0.31) | 0.243 |
| Negative affect t_-1_ | 0.04 (0.00-0.09) | 0.055 | 0.04 (-0.01-0.08) | **0.099*** |
| Mean activity stress | 0.51 (0.27-0.75) | **<0.001*** | 0.53 (0.30-0.76) | **<0.001*** |
| Mean negative affect | 0.45 (0.26-0.65) | **<0.001*** | 0.43 (0.24-0.63) | **<0.001*** |
| Age | -0.06 (-0.42-0.31) | 0.759 | -0.06 (-0.42-0.30) | 0.742 |
| Gender (male) | 1.86 (-3.83-7.54) | 0.522 | 1.99 (-3.61-7.58) | 0.486 |
| Social support (MOS Survey) | 0.32 (-2.44-3.08) | 0.820 | 0.29 (-2.42-2.99) | 0.836 |
| Child behaviour (SDQ) | -0.27 (-0.69-0.15) | 0.210 | -0.24 (-0.66-0.18) | 0.257 |
| Coping (CSES) | -0.37 (-2.17-1.43) | 0.689 | 0.02 (-1.73-1.78) | 0.980 |
| Parenting self-efficacy (MaaP) | -5.18 (-12.66-2.31) | 0.174 | -4.91 (-12.38-2.57) | 0.198 |
| Activity stress t_-1_ x Coping (CSES) | 0.02 (0.00-0.03) | **0.009*** |  |  |
| Activity stress t_-1_ x Parenting self-efficacy (MaaP) |  |  | -0.02 (-0.07-0.03) | 0.451 |

Table 5 - Models of psychosis t_0_ with Level-2 significant moderators and social stress t_-1_ with multiple imputation for missing data

|  | Model of psychosis at t_0_ with social stress t_-1_ x social ssupport | | Model of psychosis at t_0_ with social stress t_-1_ x child behaviour | | Model of psychosis at t_0_ with social stress t_-1_ x parenting self-efficacy | |
| --- | --- | --- | --- | --- | --- | --- |
|  | Estimate (95% CI) | p-value | Estimate (95% CI) | p-value | Estimate (95% CI) | p-value |
| Observation number | -0.03 (-0.05- -0.01) | **0.021*** | -0.03 (-0.05-0.00) | **0.022*** | -0.03 (-0.05-0.00) | **0.023*** |
| Social stress t_-1_ | 0.01 (-0.11-0.12) | 0.916 | 0.09 (0.04-0.14) | **0.001*** | -0.11 (-0.36-0.15) | 0.393 |
| Negative affect t_-1_ | 0.04 (0.00-0.09) | 0.059 | 0.04 (0.00-0.09) | 0.063 | 0.04 (0.00-0.09) | 0.063 |
| Mean social stress | 0.16 (-0.09-0.41) | 0.209 | 0.17 (-0.07-0.41) | 0.167 | 0.17 (-0.08-0.41) | 0.177 |
| Mean negative affect | 0.62 (0.39-0.85) | **<0.001*** | 0.62 (0.40-0.84) | **<0.001*** | 0.63 (0.40-0.85) | **<0.001*** |
| Age | 0.03 (-0.43-0.49) | 0.888 | 0.02 (-0.42-0.47) | 0.928 | 0.04 (-0.41-0.49) | 0.868 |
| Gender (male) | -0.74 (-7.61-6.14) | 0.834 | -0.41 (-7.10-6.28) | 0.904 | -0.66 (-7.41-6.09) | 0.848 |
| Social support (MOS Survey) | -1.25 (-4.67-2.17) | 0.473 | -0.94 (-4.21-2.33) | 0.574 | -0.93 (-4.25-2.39) | 0.583 |
| Child behaviour (SDQ) | -0.59 (-1.07- -0.10) | **0.017*** | -0.48 (-0.96- -0.01) | **0.048*** | -0.59 (-1.07- -0.12) | **0.014*** |
| Coping (CSES) | -0.04 (-2.13-2.04) | 0.968 | -0.07 (-2.09-1.96) | 0.947 | 0.01 (-2.02-2.05) | 0.992 |
| Parenting self-efficacy (MaaP) | -4.69 (-13.60-4.21) | 0.301 | -4.76 (-13.39-3.86) | 0.279 | -5.89 (-14.86-3.09) | 0.198 |
| Social stress t_-1_ x social support (MOS survey) | 0.01 (-0.02-0.04) | 0.375 |  |  |  |  |
| Social stress t_-1_ x child behaviour (SDQ) |  |  | 0.00 (-0.01-0.00) | 0.084 |  |  |
| Social stress t_-1_ x Parenting self-efficacy (MaaP) |  |  |  |  | 0.04 (-0.02-0.11) | 0.176 |

Table 6 - Effects of type-of-stress indicator in modelling of psychosis t_0_ with multiple imputation for missing data

|  | Model of psychosis at t_0_ with event stress t_-1_ | | Model of psychosis at t_0_ with activity stress t_-1_ | | Model of psychosis at t_0_ with social stress t_-1_ | |
| --- | --- | --- | --- | --- | --- | --- |
|  | Estimate (95% CI) | p-value | Estimate (95% CI) | p-value | Estimate (95% CI) | p-value |
| Observation number | -0.03 (-0.05- -0.01) | **0.010*** | -0.03 (-0.05- -0.01) | **0.013*** | -0.03 (-0.05- -0.01) | **0.017*** |
| Stress t_-1_ | 0.02 (-0.01-0.05) | 0.232 | 0.06 (0.02-0.10) | **0.002*** | 0.06 (0.02-0.10) | **0.004*** |
| Type-of-stress (parenting) | -0.51 (-1.99-0.97) | 0.490 | -0.63 (-1.97-0.70) | 0.350 | -1.36 (-2.62- -0.10) | **0.035*** |
| Negative affect t_-1_ | 0.05 (0.00-0.10) | 0.051 | 0.04 (0.00-0.09) | 0.062 | 0.04 (-0.01-0.09) | 0.078 |
| Mean stress | 0.30 (0.12-0.48) | **0.001*** | 0.58 (0.35-0.80) | **<0.001*** | 0.59 (0.37-0.81) | **<0.001*** |
| Mean negative affect | 0.59 (0.44-0.73) | **<0.001*** | 0.43 (0.28-0.58) | **<0.001*** | 0.42 (0.27-0.56) | **<0.001*** |
| Age | -0.06 (-0.46-0.35) | 0.776 | 0.00 (-0.34-0.34) | 0.989 | 0.00 (-0.34-0.34) | 0.994 |
| Gender (male) | 0.58 (-5.78-6.94) | 0.859 | 2.54 (-2.90-7.98) | 0.360 | 2.60 (-2.83-8.01) | 0.348 |
| Stress t_-1_ x type-of-stress (parenting) | -0.03 (-0.07-0.00) | 0.058 | -0.07 (-0.10- -0.03) | **<0.001*** | -0.02 (-0.06-0.01) | 0.192 |

## Modelling stress at t0

Table 7 - Models of stress at t_0_ with multiple imputation for missing data

|  | Model of event stress t_0_ | | Model of activity stress t_0_ | | Model of social stress t_0_ | |
| --- | --- | --- | --- | --- | --- | --- |
|  | Estimate (95% CI) | p-value | Estimate (95% CI) | p-value | Estimate (95% CI) | p-value |
| Observation number | 0.00 (-0.05-0.06) | 0.910 | 0.04 (-0.01-0.09) | 0.142 | 0.03 (-0.03-0.09) | 0.331 |
| Psychosis t_-1_ | 0.32 (0.13-0.51) | **0.002*** | 0.17 (0.02-0.33) | **0.031*** | 0.18 (-0.01-0.38) | 0.061 |
| Mean psychosis | 0.28 (0.01-0.55) | **0.042*** | 0.46 (0.26-0.65) | **0.001*** | 0.60 (0.35-0.86) | **<0.001*** |
| Age | 0.19 (-0.48-0.86) | 0.574 | -0.02 (-0.40-0.37) | 0.927 | -0.24 (-0.78-0.31) | 0.400 |
| Gender (male) | -0.60 (-11.09-9.89) | 0.911 | -2.80 (-8.92-3.32) | 0.370 | -1.30 (-9.71-7.11) | 0.762 |

Table 8 - Models of stress at t_0_ with Level 2 variables included as covariates with multiple imputation for missing data

|  | Model of event stress t_0_ | | Model of activity stress t_0_ | | Model of social stress t_0_ | |
| --- | --- | --- | --- | --- | --- | --- |
|  | Estimate (95% CI) | p-value | Estimate (95% CI) | p-value | Estimate (95% CI) | p-value |
| Observation number | 0.00 (-0.05-0.06) | 0.912 | 0.04 (-0.01-0.09) | 0.142 | 0.03 (-0.03-0.09) | 0.330 |
| Psychosis t_-1_ | 0.32 (0.13-0.51) | **0.002*** | 0.17 (-0.02-0.33) | **0.031*** | 0.18 (-0.01-0.38) | 0.062 |
| Mean psychosis | 0.10 (-0.23-0.43) | 0.533 | 0.51 (0.28-0.73) | **<0.001*** | 0.51 (0.19-0.83) | **0.002*** |
| Age | 0.20 (-0.47-0.87) | 0.553 | 0.07 (-0.34-0.47) | 0.754 | -0.37 (-0.96-0.21) | 0.207 |
| Gender (male) | -0.78 (-11.22-9.66) | 0.884 | -4.01 (-10.36-2.34) | 0.216 | -0.15 (-8.54-8.25) | 0.973 |
| Social support (MOS Survey) | -2.98 (-8.05-2.09) | 0.249 | -1.25 (-4.30-1.81) | 0.423 | 3.08 (-1.54-7.70) | 0.190 |
| Child behaviour (SDQ) | -0.03 (-0.74-0.68) | 0.939 | -0.21 (-0.63-0.22) | 0.341 | 0.29 (-0.34-0.91) | 0.367 |
| Coping (CSES) | -1.71 (-4.76-1.35) | 0.273 | 0.13 (-1.64-1.89) | 0.887 | -0.96 (-3.37-1.45) | 0.433 |
| Parenting self-efficacy (MaaP) | -3.95 (-18.83-10.94) | 0.602 | 2.68 (-5.72-11.09) | 0.531 | -2.94 (-16.61-10.74) | 0.669 |

Table 9 - Model of event stress t_0_ with Level-2 significant moderators with multiple imputation for missing data

|  | Model of event stress t_0_ with psychosis t_-1_ x coping | |
| --- | --- | --- |
|  | Estimate (95% CI) | p-value |
| Observation number | 0.00 (-0.05-0.06) | 0.917 |
| Psychosis t_-1_ | 0.38 (-0.01-0.78) | 0.057 |
| Mean psychosis | 0.09 (-0.25-0.43) | 0.594 |
| Age | 0.21 (-0.46-0.88) | 0.537 |
| Gender (male) | -0.93 (-11.37-9.52) | 0.862 |
| Social support (MOS Survey) | -3.06 (-8.13-2.01) | 0.236 |
| Child behaviour (SDQ) | -0.04 (-0.74-0.67) | 0.914 |
| Coping (CSES) | -1.35 (-4.86-2.16) | 0.451 |
| Parenting self-efficacy (MaaP) | -4.23 (-19.00-10.53) | 0.572 |
| Psychosis t_-1_ x Coping (CSES) | -0.01 (-0.08-0.05) | 0.701 |

Table 10 - Model of activity stress t_0_ with Level-2 significant moderators with multiple imputation for missing data

|  | Model of activity stress t_0_ with psychosis t_-1_ x gender | |
| --- | --- | --- |
|  | Estimate (95% CI) | p-value |
| Observation number | 0.04 (-0.02-0.09) | 0.152 |
| Psychosis t_-1_ | 0.20 (0.03-0.37) | **0.021*** |
| Mean psychosis | 0.52 (0.29-0.74) | **<0.001*** |
| Age | 0.06 (-0.35-0.46) | 0.789 |
| Gender (male) | -1.34 (-8.89-6.21) | 0.727 |
| Social support (MOS Survey) | -1.15 (-4.24-1.95) | 0.467 |
| Child behaviour (SDQ) | -0.22 (-0.65-0.21) | 0.314 |
| Coping (CSES) | 0.06 (-1.73-1.85) | 0.947 |
| Parenting self-efficacy (MaaP) | 3.94 (-4.54-12.42) | 0.362 |
| Psychosis t_-1_ x Gender (male) | -0.13 (-0.33-0.06) | 0.180 |

Table 11 - Models of social stress t_0_ with Level-2 significant moderators with multiple imputation for missing data

|  | Model of social stress t_0_ with psychosis t_-1_ x child behaviour | | Model of social stress t_0_ with psychosis t_-1_ x parenting self-efficacy | | Model of social stress t_0_ with psychosis t_-1_ x gender | |
| --- | --- | --- | --- | --- | --- | --- |
|  | Estimate (95% CI) | p-value | Estimate (95% CI) | p-value | Estimate (95% CI) | p-value |
| Observation number | 0.03 (-0.03-0.09) | 0.342 | 0.03 (-0.03-0.09) | 0.365 | 0.03 (-0.03-0.09) | 0.361 |
| Psychosis t_-1_ | 0.07 (-0.20-0.35) | 0.585 | 1.00 (0.21-1.78) | **0.013*** | 0.24 (0.03-0.46) | **0.026*** |
| Mean psychosis | 0.52 (0.21-0.84) | **0.002*** | 0.48 (0.16-0.80) | **0.004*** | 0.53 (0.22-0.85) | **0.001*** |
| Age | -0.30 (-0.88-0.29) | 0.315 | -0.34 (-0.88-0.20) | 0.213 | -0.39 (-0.95-0.17) | 0.168 |
| Gender (male) | -0.34 (-8.43-7.76) | 0.935 | 0.64 (-6.98-8.26) | 0.869 | 5.32 (-4.93-15.57) | 0.307 |
| Social support (MOS Survey) | 2.84 (-1.75-7.42) | 0.222 | 2.31 (-1.98-6.59) | 0.289 | 3.27 (-1.20-7.74) | 0.150 |
| Child behaviour (SDQ) | -0.05 (-0.83-0.72) | 0.892 | 0.27 (-0.30-0.84) | 0.352 | 0.26 (-0.35-0.86) | 0.406 |
| Coping (CSES) | -0.91 (-3.23-1.41) | 0.440 | -1.19 (-3.42-1.05) | 0.298 | -1.11 (-3.44-1.22) | 0.350 |
| Parenting self-efficacy (MaaP) | -1.75 (-15.72-12.23) | 0.803 | 5.36 (-9.61-20.33) | 0.477 | -0.33 (-13.20-12.54) | 0.960 |
| Psychosis t_-1_ x Child behaviour (SDQ) | 0.01 (0.00-0.03) | 0.182 |  |  |  |  |
| Psychosis t_-1_ x Parenting Self-Efficacy (MaaP) |  |  | -0.22 (-0.43- -0.01) | **0.038*** |  |  |
| Psychosis t_-1_ x Gender (male) |  |  |  |  | -0.27 (-0.57-0.03) | 0.072 |
