# Supplementary material for: Parenting and psychosis: An experience sampling methodology study investigating the inter‐relationship between stress from parenting and positive psychotic symptoms
Source: Br J Clin Psychol. 2022 Aug 8;61(4):1236–58. doi: 10.1111/bjc.12389 (PMC9804428; doi:10.1111/bjc.12389)
Supplement: Supplementary file 3 — Appendix S3 [file BJC-61-1236-s001.docx]

# Exploratory analyses of temporal relationship and autocorrelation

Table 1 - Models of psychosis t_0_ including parenting stress at t_0_ and t_-1_ to investigate the temporal relationship and psychosis t_-1_ to investigate autocorrelation

|  | Model of psychosis at t_0_ with parenting event stress t_-1_ | | Model of psychosis at t_0_ with parenting activity stress t_-1_ | | Model of psychosis at t_0_ with parenting social stress t_-1_ | |
| --- | --- | --- | --- | --- | --- | --- |
|  | Estimate (95% CI) | p-value | Estimate (95% CI) | p-value | Estimate (95% CI) | p-value |
| Observation number | 0.00 (-0.06-0.06) | 0.919 | -0.04 (-0.11-0.02) | 0.209 | -0.05 (-0.08- -0.01) | **0.018*** |
| Parenting stress t_0_ | 0.18 (0.11-0.25) | **<0.001*** | 0.10 (0.01-0.20) | **0.037*** | 0.06 (0.02-0.09) | **0.001*** |
| Parenting stress t_-1_ | -0.01 (-0.08-0.07) | 0.855 | 0.03 (-0.07-0.13) | 0.577 | -0.01 (-0.04-0.03) | 0.696 |
| Psychosis t_-1_ | 0.29 (0.13-0.45) | **<0.001*** | 0.46 (-0.27-0.65) | **<0.001*** | 0.29 (0.19-0.38) | **<0.001*** |
| Negative affect t_-1_ | -0.13 (-0.27-0.01) | 0.065 | -0.13 (-0.27-0.01) | 0.079 | -0.02 (-0.11-0.07) | 0.654 |
| Mean stress | 0.18 (0.01-0.34) | **0.041*** | 0.22 (0.02-0.43) | **0.035*** | 0.23 (0.08-0.38) | **0.003*** |
| Mean negative affect | 0.60 (0.44-0.76) | **<0.001*** | 0.39 (0.18-0.59) | **<0.001*** | 0.40 (0.26-0.53) | **<0.001*** |
| Age | -0.03 (-0.37-0.30) | 0.818 | -0.15 (-0.53-0.23) | 0.395 | -0.03 (-0.36-0.31) | 0.874 |
| Gender (male) | 3.55 (-2.52-9.63) | 0.188 | 4.17 (-1.90-10.25) | 0.164 | 2.98 (-2.47-8.44) | 0.271 |

Table 2 - Models of parenting stress t_0_ including psychosis at t_0_ and t_-1_ to investigate the temporal relationship and parenting stress t_-1_ to investigate autocorrelation

|  | Model of parenting event stress t_0_ | | Model of parenting activity stress t_0_ | | Model of parenting social stress t_0_ | |
| --- | --- | --- | --- | --- | --- | --- |
|  | Estimate (95% CI) | p-value | Estimate (95% CI) | p-value | Estimate (95% CI) | p-value |
| Observation number | -0.05 (-0.17-0.06) | 0.361 | 0.00 (-0.14-0.14) | 0.979 | -0.02 (-0.13-0.09) | 0.751 |
| Psychosis t_0_ | 0.73 (0.44-1.02) | **<0.001*** | 0.69 (0.22-1.16) | 0.005 | 0.46 (0.16-0.76) | **0.003*** |
| Psychosis t_-1_ | 0.13 (-0.18-0.43) | 0.407 | 0.15 (-0.28-0.58) | 0.495 | 0.09 (-0.19-0.37) | 0.540 |
| Parenting stress t_-1_ | 0.31 (0.17-0.45) | **<0.001*** | 0.34 (0.15-0.52) | 0.001 | 0.23 (0.13-0.33) | **<0.001*** |
| Mean psychosis | -0.40 (-0.81-0.00) | 0.052 | -0.42 (-0.93-0.09) | 0.107 | 0.05 (-0.33-0.42) | 0.809 |
| Age | -0.23 (-0.97-0.51) | 0.513 | 0.29 (-0.14-0.73) | 0.181 | -0.06 (-0.57-0.45) | 0.797 |
| Gender (male) | -9.35 (-22.32-3.63) | 0.140 | -10.87 (-18.84- -2.90) | 0.009 | -8.52 (-16.90- -0.14) | **0.047*** |
